# Lectures on Military Surgery

**Published:** 1861

**Authors:** E. Andrews

**Affiliations:** Professor of Surgery, Surgeon to the Mercy Hospital; to the Lind Medical Dispensary, &c.


### LECTURES ON MILITARY SURGERY:

### DELIVERED DURING THE SUMMER COURSE OF THE MEDICAL DEPARTMENT OF LIND UNIVERSITY.

By E. ANDREWS, A. M., M. D.,

PROFESSOR OF SURGERY; SURGEON TO THE MERCY HOSPITAL; TO THE LIND MEDICAL DISPENSARY, &C.

### LECTURE FIRST.

War always contemplates the destruction of human life; but, in all civilized nations, measures are taken to alleviate the horrors of combat by attention to the condition of the sick and the wounded. Hence arises the necessity of a medical department in all modern armies.

Among the ancients, we find but faint traces of any systematic organization of military surgery; still, the existence of such a service in the armies of old, and the high value set upon it, is obvious, even from the poetry of Homer.

The objects of a medical department are three in number:

I. To preserve the health of the army, in order that there shall be as many men as possible, at all times, in a condition to fight.

II. To keep up the courage of the troops. Men are reluctant to rush into a fight, if they know that in case of being wounded, they will not be cared for, but left to die on the field.

III. To fulfil the duties of humanity, which require that the terrors of war shall be as much as possible diminished.

In the United States Army, this branch of the service is called the "Medical Department," but the officers of it are denominated Surgeons. Although these men are officially called Surgeons, it must not be supposed that their business is chiefly surgery. During the times of peace their services are almost exclusively medical, and even in war, the wounded are few in number compared with those sick of disease. During the Mexican war, the deaths from disease in our army were seven times as many as the deaths from wounds. Other wars show similar statistics.

The mode of admission to the Medical Department of the army is as follows: For permission to present himself before the Examining Board, application must be made to the Secretary of War, stating the present residence, date and place of birth of the applicant; this application must be accompanied by respectable testimonials of his professional, moral and physical qualifications; and further, the applicant must be between the ages of twenty-one and twenty-five years. It is not requisite that he should be a graduate of any college, as the examiners trust entirely to the results of their own questioning, and give no credit to the testimonial of a diploma. If the Secretary of War grants the request, the applicant will receive an invitation to appear at a certain time and place for an examination. The Board of Examiners generally consists of three surgeons, appointed from time to time, and who meet, sometimes in one city and sometimes in another, as often as required, for the examination of candidates. It is the object of the Government to secure a superior class of men; hence the examination is very rigid, embracing both the literary and professional qualifications of the candidate. Those who are approved, are notified to return to their homes and await orders, and are called into service as fast as required.

On first entering the army, the medical officers have the title of Assistant Surgeons. This, however, is by no means a well chosen term, insomuch as they are not the mere *assistants* of the Surgeons in the ordinary sense, but simply *Surgeons* of the lowest grade. The Assistant Surgeons have the rank of Lieutenant. After five years of service, they again pass before a Board of Examiners. If found deficient in knowledge, they are dropped from the service; but if not, they are promoted to the grade of "Assistant Surgeons of more than five years service," and are provided with increased pay. After another five years they are again promoted, and in twelve or fifteen years may attain the rank of Surgeon. The Surgeon has the rank of Captain.

At the head of the whole Medical Department is the Surgeon-General, (Dr. Lawson is the present incumbent). The Surgeon-General resides at Washington, and has control of all the matters pertaining to his branch of the service. This position is the highest one attainable, and is generally reached according to the rule of seniority, so that a man can always tell exactly how many men must die before he can reach the highest position.

The pay of the medical officers is as follows:

Surgeon-General, $2,740 a year.

| | Pay per Month. | Allowance for servants, rations, and horses. | Total per Month. |
|---|---|---|---|
| Surgeons of over 10 years service, | $80 00 | $143 00 | $223 00 |
| " less than " " | 80 00 | 107 00 | 187 00 |
| Assis't Surgeons of over 10 years service, | 70 00 | 103 50 | 173 50 |
| " " " 5 " | 70 00 | 67 50 | 137 50 |
| " " less than 5 " | 53 33 | 67 50 | 120 83 |

In case of the officer being stationed where servants, horses, and extra rations are not required, an equivalent for servants' rations and forage for horses may be drawn in money.

In the volunteer forces lately raised by requisition on the States, the rank and pay is the same as in the regular army, but the mode of admission varies according to the law of the States. In Illinois there is a Board of Examiners, consisting of Dr. N. S. Davis, of Chicago; Dr. G. W. Stipp, of Bloomington; Dr. Wm. M. Chambers, of Charleston; Dr. Chas. Ryan, of Springfield; and Dr. Carpenter, of St. Clair County.

All candidates who choose, appear before this Board, and if competent, are recommended for Surgeons or Assistant Surgeons. From the list of persons thus approved, the Colonels of the regiments select one Surgeon and one Assistant Surgeon for each regiment.*

In Wisconsin, the proper authorities select without examination, such surgeons as they consider suitable.

The Surgeons and Assistant Surgeons are provided by Government with a full assortment of surgical instruments, medicines, and a moderate list of the most necessary books. In certain circumstances, private physicians are temporarily employed by the army, at from thirty to one hundred dollars a month.

The duties of an army surgeon are various, but may be summed up under three heads: 1st, The examination of new recruits; 2d, The arranging and advising about the sanitary care of the army; 3d, The care of the Hospitals and Ambulances.

In examining new recruits the object is to ascertain whether the men are sound and physically competent for service. For this purpose the surgeon must take the recruit into a private room and cause him to strip completely. He then examines his chest, his limbs, and, in fact, his whole body. The recruit must not be below a certain regulation stature. His eyesight must be good, the front teeth, (required in biting cartridges)

---

* This applies only to the six regiments just raised. Colonels of regiments appoint their own Surgeons and Assistant Surgeons for the regiments now organizing, without reference to certificates of qualifications. [See editorial on a preceding page. R.]

must be present; the lungs and heart must be sound; the skin must be free from contagious eruptions; there must be no hernia; and the limbs must be free from ulcers, extensive cicatrices, or any other blemish which would impair their use. Deafness, or any other serious disease, is also ground for rejecting the applicant. The recruiting regulations of different nations, are different. In France it used to be remarked that the rules were rather framed to prevent men who should serve, from evading the duty; while in England and the United States the object seems to be rather to prevent any but the best men from being accepted. In general, therefore, the more anxious a government is to obtain men, the lower will be its standard of quality as given for the guidance of the examining surgeon. In case an involuntary levy of men is made in any community, the surgeon will meet a certain number of feigned diseases, gotten up by the recruit to prevent his enrolment. In these cases, it is necessary for the Surgeon to use all his acuteness and caution lest on the one hand the Government be defrauded of proper men, or on the other it be saddled with useless invalids.

The attempt, in ancient times, to escape military service by surgical mutilation has transmitted a peculiar word even to our times. When the chief arm of service was the bow, the thumb of the right hand was necessary in drawing the string with the arrow. Men who desired to escape the dangers of war therefore cut off their thumb, and thus rendered themselves unable to fight. Hence *pollicé truncata*, or cut thumb, was the term applied to such cowards. This phrase, transmitted through the French language, became first, *pol tronc*, and afterwards in English, *poltroon.*

Considerable debate has been excited respecting the relative rank and authority of medical and military officers. Formerly, medical men had no established rank in any civilized army. In France, the Surgeon cannot order even the nurse to obey him, he being supposed only to examine, prescribe, and operate. In England, the medical men have authority, but no rank. In this country they have both rank and authority. The Assistant Surgeons rank as Lieutenants, and the Surgeons as Captains. Even this late improvement has been a matter of hot dispute. It is contended on the one hand, that to permit the Surgeons to control exclusively the matters of their own department, will set up an independent power in the army, which would come in collision with military plans and prevent efficiency of service. On the other hand it is urged that Surgeons are alone competent to direct in sanitary and medical affairs, and that to refuse them the authority to execute their plans, is to imperil the army by pestilence, and in many instances, to insure its actual failure and disgrace—things which have occurred from this cause many times in history. As to rank, it is claimed that Surgeons on the field of battle, are often exposed to fire, and off from it they face, day by day, the greater dangers of pestilence in crowded hospitals; they are therefore deserving of the reward and stimulus of suitable rank.

In this dispute, the true position appears to be this: First as to executive authority. The chief end of the army is to gain the battle, the campaign, and the war. To this end, in the very nature of military affairs, all questions of life, health and property are subordinate; hence the field officer must be allowed to march his troops into pestilential marshes. If the site of the Hospital is required for a redoubt or a battery, he must have power to remove it, and in all cases, he must be allowed for military reasons, to execute military operations, even though the sanitary interests of the army be, for the time, injured. But in the internal affairs of the Medical Department the Surgeon should have power to enforce his own authority.

In respect to rank, there is no reason why the medical officers should not be put on an equality with those of other branches of the service. Certainly, their knowledge, their skill, the dangers which they incur, and the importance of their functions entitle them to rank with the best.

---

John C. Dalton, Jr., M. D., the eminent physiologist, has been appointed Assistant Surgeon to the Seventh New York Regiment.

